# Entwicklung und Validierung des Essen Melanoma Quality of Life Inventory (EMQoLI)

**DOI:** 10.1111/ddg.15998_g

**Published:** 2026-09-08

**Authors:** Venja Musche, Theresa Schadendorf, Adam Schweda, Georg Lodde, Jessica Neumann, Lisa Zimmer, Svea Hüning, Dirk Schadendorf, Eva‐Maria Skoda, Elisabeth Livingstone, Martin Teufel

**Affiliations:** ^1^ Klinik für Psychosomatische Medizin und Psychotherapie LVR‐Universitätsklinik Essen Universität Duisburg‐Essen, Essen; ^2^ Zentrum für Translationale Neuro‐ und Verhaltenswissenschaften (C‐TNBS) Universität Duisburg‐Essen, Essen; ^3^ Deutsches Konsortium für Translationale Krebsforschung (DKTK) Campus Essen & Nationales Centrum für Tumorerkrankungen (NCT‐West) Campus Essen & Forschungsallianz Ruhr Forschungszentrum One Health Campus Essen; ^4^ Klinik für Dermatologie Universitätsklinikum Essen; ^5^ Klinik für Dermatologie Klinikum Dortmund

**Keywords:** Befragungen und Fragebögen, gesundheitsbezogene Lebensqualität, Krebs, malignes Melanom, Psychometrie, Cancer, Melanoma, Psychometrics, Quality of Life, Surveys and Questionnaires

## Abstract

**Hintergrund und Ziele:**

Neue Behandlungsformen für das Melanom, wie zielgerichtete Therapien und Immuntherapien, bringen im Vergleich zu traditionellen Verfahren wie Operation und Chemotherapie spezifische Nebenwirkungen mit sich. Bestehende Instrumente zur Erfassung der gesundheitsbezogenen Lebensqualität (HRQoL) berücksichtigen diese neuen Therapieeffekte oft unzureichend und fokussieren primär auf Chemotherapie und chirurgische Eingriffe. Ziel dieser Studie war die Entwicklung und Validierung eines neuen HRQoL‐Instruments für Melanompatient aller Tumorstadien, das ein breiteres Spektrum an Nebenwirkungen abdeckt.

**Patienten und Methodik:**

Das *Essen Melanoma Quality of Life Inventory* (EMQoLI) wurde an 387 deutschsprachigen Patienten mit malignem Melanom aller Tumorstadien getestet.

**Ergebnisse:**

Eine explorative Faktorenanalyse ergab eine Drei‐Faktoren‐Struktur. Acht Items wurden aufgrund niedriger oder mehrfacher Ladungen ausgeschlossen, sodass die finale Version aus 25 Items mit zufriedenstellender Reliabilität und interner Konsistenz besteht. Korrelationsanalysen zeigten eine gute konvergente und diskriminante Validität.

**Schlussfolgerungen:**

Das EMQoLI ist ein deutschsprachiges Selbstberichtsverfahren mit 25 Items auf einer fünfstufigen Likert‐Skala, das die HRQoL von Melanompatient:innen aller Tumorstadien erfasst. Es bietet Fachkräften im Gesundheitswesen ein effizientes Instrument zur Identifikation von Patient mit psychosozialem Unterstützungsbedarf und kann als standardisiertes Messinstrument im klinischen Kontext eingesetzt werden.

## EINLEITUNG

Das Melanom war im Jahr 2020 für rund 57 000 Todesfälle verantwortlich.^1^ Etwa 325 000 neue Fälle wurden im Jahr 2020 diagnostiziert, und demografische Prognosen deuten darauf hin, dass die Inzidenz bis 2040 um über 50 % steigen wird.^1^ In den letzten Jahrzehnten haben sich die Behandlungsmöglichkeiten vom chirurgischen Entfernen, der Strahlentherapie und konventionellen Chemotherapien hin zu zielgerichtete Therapie und Immuntherapien entwickelt.^2^, ^3^, ^4^ Obwohl diese neuen Therapien Symptome lindern, den Krankheitsverlauf verlangsamen und letztlich die Überlebensraten bei Melanom im Spätstadium verbessern, sind sie auch mit unterschiedlichen Toxizitätsprofilen und schwerwiegenden therapiebedingten Nebenwirkungen (TRAE) verbunden wie Kolitis, Hepatitis, Hypophysitis, endokrinen Erkrankungen, kardiotoxischen oder kutanen Nebenwirkungen sowie Retinopathien.^2^, ^5^, ^6^, ^7^, ^8^ Zudem erhalten viele Patienten diese Behandlungen inzwischen adjuvant, also in einem Krankheitsstadium mit geringerem Risiko für ein Rezidiv oder Melanom‐bedingten Tod. Dies erfordert eine veränderte Perspektive auf die Krankheits‐ und Therapiebelastung.

Auch nach chirurgischer Behandlung verbleiben Melanompatienten über Jahre hinweg in einem erhöhten Rückfallrisiko sowie Risiko für Metastasen oder Krankheitsprogression, was eine kontinuierliche Nachsorge erforderlich macht.^2^, ^9^ Die Auswirkungen eines Melanoms hängen zudem vom Tumorstadium ab: Frühstadien mit dünnen Melanomen benötigen oft nur eine Operation und eine Nachsorge, die aus regelmäßigen Hautuntersuchungen und Lymphknotenabtastungen besteht.^2^, ^10^, ^11^ Im Gegensatz dazu müssen Patienten mit fortgeschrittenem Stadium und signifikantem Rückfallrisiko regelmäßig bildgebende Diagnostik wahrnehmen und wachsam auf mögliche Rückfälle achten.^2^, ^9^, ^10^, ^11^ Patient im fortgeschrittenen Stadium können ebenfalls Symptome von Metastasen erfahren und unter den Nebenwirkungen systemischer Behandlungen leiden.^2^ Trotz therapeutischer Fortschritte sind viele Melanompatienten weiterhin zusätzlichen Belastungen ausgesetzt, darunter finanzielle und soziale Sorgen. Das Melanom muss somit als chronische, potenziell lebensbedrohliche Erkrankung betrachtet werden, die die gesundheitsbezogene Lebensqualität (health‐related quality of life, HRQoL) erheblich beeinträchtigt.^7^, ^12^, ^13^

Die HRQoL ist ein multidimensionales Konstrukt. Es umfasst physische und soziale Funktionen als Gesundheitskomponenten sowie psychologische und verhaltensbezogene Aspekte des Wohlbefindens und der Lebensqualität, wie sie durch Krankheit oder Behandlung beeinflusst werden.^14^, ^15^, ^16^ Als patientenberichtetes Ergebnismaß hat die Erfassung der HRQoL in der Gesundheits‐ und Krebsforschung erheblich an Bedeutung gewonnen.^16^, ^17^ HRQoL‐Fragebögen liefern subjektive Informationen, die medizinische Parameter sinnvoll ergänzen – insbesondere bei Erkrankungen, in denen therapiebedingte Nebenwirkungen und Langzeitüberleben die Lebensqualität und Funktionsfähigkeit der Patienten stark beeinflussen können.^17^ Daher sollte die HRQoL‐Erfassung fester Bestandteil von Diagnose, Nachsorge und klinischen Studien bei Melanompatienten sein, um die subjektiven Auswirkungen von Erkrankung und Behandlung zur medizinischen Entscheidungsfindung nutzbar zu machen.^17^

Zahlreiche Instrumente zur HRQoL existieren bereits.^17^ Diese sind jedoch meist generisch und nicht spezifisch für Krebspatienten oder Melanompatienten. Melanom‐spezifische Aspekte wie Tumorstadium, Art der Therapie, Nebenwirkungen oder sonnenvermeidendes Verhalten werden in generischen HRQoL‐Maßen nicht berücksichtigt. Daher spiegeln bestehende HRQoL‐Instrumente die Melanom‐spezifische Lebensqualität nur unzureichend wider.^5^, ^18^, ^19^, ^20^ Studien zu Melanompatienten verwenden häufig krebsbezogene HRQoL‐Fragebögen wie den *European Organisation for Research and Treatment of Cancer Quality of Life Questionnaire* (EORTC QLQ‐C30).^19^, ^20^, ^21^, ^22^ Die HRQoL‐Messung kann jedoch auch durch Faktoren beeinflusst sein, die über klassische Messgrößen wie Krankheitsverlauf und Therapieerfolg hinausgehen – etwa durch mangelndes gesellschaftliches Verständnis und Anerkennung des Melanoms.^5^, ^18^, ^19^, ^20^ Selbst krebsspezifische HRQoL‐Fragebögen könnten daher nicht sensitiv genug sein, um Melanom‐spezifische Aspekte zu erfassen.

Nur wenige HRQoL‐Instrumente sind speziell für Melanompatienten entwickelt worden. Auf Grundlage des EORTC QLQ‐C30 wurde das *EORTC Melanoma Module* (QLQ‐MEL38) entwickelt,^19^ später überarbeitet durch den *Melanoma Concerns Questionnaire* (MCQ‐28) im Jahr 2019.^7^ Die Validierung dieser Instrumente wurde jedoch kritisiert.^7^, ^19^ Das am häufigsten verwendete Melanom‐spezifische HRQoL‐Instrument ist die *Functional Assessment of Cancer Therapy‐Melanoma Subscale* (FACT‐M).^23^, ^24^ Eine Überarbeitung der Struktur und Antwortformate der FACT‐M wurde immer wieder empfohlen.^7^, ^25^ Studien zeigen zudem, dass eine niedrigere HRQoL mit psychischer Belastung und unzureichender Versorgung einhergeht – Aspekte, die in der FACT‐M nicht berücksichtigt werden.^5^, ^26^ Ursprünglich wurde die FACT‐M für klinische Studien an Patienten mit fortgeschrittenem Melanom entwickelt, mit höherer Mortalität und intensiverer Therapie.^27^ Aktuelle HRQoL‐Instrumente konzentrieren sich vor allem auf Nebenwirkungen von Chemo‐ und chirurgischer Therapie und erfassen möglicherweise nicht vollständig die spezifischen Nebenwirkungen moderner Therapien wie Immun‐ oder zielgerichteter Therapie.^5^, ^28^

Zusammenfassend lassen sich bestehende Instrumente zur Erfassung der HRQoL nicht ausreichend auf Melanom‐spezifische Aspekte anwenden – darunter Symptome, Tumorstadien, therapiebedingte Belastung oder psychologische Belastung.^2^, ^5^, ^26^, ^28^, ^29^, ^30^ Um diese Lücke zu schließen, wurde ein neues Instrument zur HRQoL‐Erfassung beim Melanom entwickelt: das *Essen Melanoma Quality of Life Inventory* (EMQoLI). Ziel der vorliegenden Studie war es, die Struktur und psychometrischen Eigenschaften des neuen Instruments an einer großen Stichprobe von Patienten mit unterschiedlichem Tumorstadium zu untersuchen.

## PATIENTEN UND METHODIK

### Teilnehmenden und Studiendesign

Die Teilnehmenden wurden von Dezember 2019 bis Januar 2023 in den dermatologischen Abteilungen des Universitätsklinikums Essen und des Klinikums Dortmund sowie in Online‐Selbsthilfegruppen für Melanompatienten rekrutiert. Die Studie basierte auf einer umfassenden Online‐Umfrage, die mehrere standardisierte Fragebögen sowie selbst erstellte Fragen umfasste und über die Plattform Unipark (Tivian XI GmbH, Berlin) durchgeführt wurde. Die Umfrage war ein zentraler Bestandteil der Studie und ging über den Rahmen des EMQoL hinaus, das im Mittelpunkt dieses Artikels steht. Alle Teilnehmenden gaben vor ihrer freiwilligen Teilnahme an der anonymen Selbstberichtsstudie eine elektronische Einverständniserklärung ab. Einschlusskriterien waren *(1)* eine gesicherte Melanomdiagnose, *(2)* ein Alter von mindestens 18 Jahren und *(3)* ausreichende Deutschkenntnisse. Die endgültige Stichprobe umfasste 387 Teilnehmende. Die durchschnittliche Dauer der gesamten Umfrage betrug 26 Minuten und 46 Sekunden (Standardabweichung [SD] = 11:59). Die Querschnittsstudie wurde von der Ethikkommission der Medizinischen Fakultät der Universität Duisburg‐Essen genehmigt (19‐8862‐BO).

### Entwicklung des EMQoLI

Die aktuelle Literatur zur gesundheitsbezogenen Lebensqualität (HRQoL) von Melanompatienten, bestehende Messinstrumente sowie das Feedback eines multiprofessionellen Expertengremiums, bestehend aus Pflegekräften und Ärzten mit Erfahrung in der Betreuung von Melanompatienten, wurden bei der Item‐Erstellung berücksichtigt. Alle Items wurden auf Deutsch formuliert. Vor der Hauptstudie wurde eine Pilotstudie (n = 59 Melanompatienten unterschiedlicher Tumorstadien) durchgeführt, um die Qualität der Items, Akzeptanz, Themenabdeckung und Länge des Fragebogens zu bewerten (siehe ergänzendes Material im Online‐Supplement). Basierend auf den Ergebnissen wurde die Länge des Fragebogens angepasst und einzelne Items zur besseren Verständlichkeit umformuliert. Die endgültigen Items wurden nach mehreren Feedbackrunden im Expertenteam einvernehmlich ausgewählt. Das Expertenteam bestand aus Psychologen mit Erfahrung in Testkonstruktion sowie auf Dermatoonkologie spezialisierten Ärzten und Pflegekräften. Die vorläufige Version des EMQoLI umfasste 33 Items, die auf einer fünfstufigen Likert‐Skala von 0 = „stimme überhaupt nicht zu“ bis 4 = „stimme voll und ganz zu“ beantwortet wurden, wobei höhere Werte eine niedrigere Lebensqualität anzeigen. Sieben Items wurden invertiert, um Antwortverzerrungen zu reduzieren und die Datenqualität zu verbessern. Die Instruktionen des Fragebogens gaben an, die Items mit Bezug auf die letzten zwei Wochen zu beantworten. Das Ausfüllen des EMQoLI dauert durchschnittlich etwa fünf Minuten. Die finale deutsche Version des EMQoLI sowie eine vorläufige englische Version sind im ergänzenden Online‐Material verfügbar.

### Studienvariablen

Soziodemografische und medizinische Informationen wurden zur Beschreibung der Stichprobe erfasst. Zur Überprüfung der konvergenten und diskriminanten Validität des EMQoLI – also seiner Übereinstimmung mit verwandten und seiner Abgrenzung von nichtverwandten Messinstrumenten – wurden folgende Instrumente eingesetzt:

*European Organization for Research and Treatment of Cancer Core Quality of Life Questionnaire (EORTC‐QLQ‐C30)*.^20^ Das EORTC QLQ‐C30 ist ein validiertes, multidimensionales Instrument zur Erfassung der Lebensqualität bei onkologischen Patienten. Es enthält Funktionsskalen, Symptomskalen, eine Skala zur allgemeinen Lebensqualität sowie Einzelitems. Es wird erwartet, dass zwischen dem neu entwickelten EMQoLI und dem EORTC QLQ‐C30 ein signifikanter positiver Zusammenhang besteht.

*Hospital Anxiety and Depression Scale (HADS)*.^31^ Die HADS ist ein validiertes Screening‐Instrument zur Erfassung von Angst‐ und Depressionssymptomen, bestehend aus jeweils sieben Items. Es wird angenommen, dass Angst und Depression mit einer beeinträchtigten, melanombezogenen Lebensqualität einhergehen, was sich in einem positiven Zusammenhang mit dem EMQoLI widerspiegeln sollte.

*Generalized Self‐Efficacy Scale (GSE)*.^32^ Die deutsche Version der GSE besteht aus zehn Items, die auf einer vierstufigen Likert‐Skala bewertet werden. Sie ist ein validiertes Selbstbeurteilungsinstrument zur Erfassung von Selbstwirksamkeit und optimistischen Selbstüberzeugungen im Umgang mit schwierigen Lebenssituationen. Hier wird ein starker negativer Zusammenhang mit dem EMQoLI erwartet.

*Barratt Impulsiveness Scale – Kurzversion (BIS‐15)*.^33^ Die BIS‐15 umfasst 15 Items, die auf einer vierstufigen Likert‐Skala beantwortet werden, und misst drei Dimensionen: nicht‐planende Impulsivität, motorische Impulsivität und aufmerksamkeitsbezogene Impulsivität. Hier wird ein nicht signifikanter oder geringer Zusammenhang mit dem EMQoLI erwartet.

### Datenanalyse

Die Datenanalyse wurde mit R Version 4.4.1,^34^ durchgeführt. Da es sich um die erste Validierungsstudie des EMQoLI handelt, wurde ein explorativer faktorenanalytischer Ansatz zur Testkonstruktion und Extraktion der entsprechenden Subskalen gewählt. Hierbei kam der Befehl *n_factors* aus dem R‐Paket *parameters*,^35^ zum Einsatz, der die Anwendung verschiedener Verfahren zur Bestimmung der optimalen Faktorenzahl (zum Beispiel Parallelanalyse, Velicer's MAP) automatisiert. Anschließend wurden *explorative Faktorenanalysen* (EFA) nach Standardverfahren des R‐Pakets *psych*,^36^ durchgeführt, unter der Verwendung einer Varimax‐Rotation basierend auf polychorischen Korrelationsmatrizen. Die Itemauswahl erfolgte anhand der Faktorladungen, wobei ein Cutoff‐Wert von 0,4 definiert wurde, sowie anhand der Kommunalitäten der Items (Cut‐off: < 0,25). Für das endgültige Instrument wurde MacDonald's Omega als Maß für die Reliabilität berichtet. Spearman‐Korrelationskoeffizienten wurden zur Darstellung der konvergenten beziehungsweise diskriminanten Validität verwendet. Um Unterschiede in den Testergebnissen in krankheitsspezifischen und soziodemografischen Kategorien (zum Beispiel Tumorstadium, Geschlecht) zu untersuchen, wurden Mann‐Whitney‐U‐Tests sowie Kruskal‐Wallis‐Tests durchgeführt. Die Stichprobengröße von 387 Teilnehmenden entspricht den Empfehlungen für Validierungsstudien.^37^

## ERGEBNISSE

### Stichprobenmerkmale

Insgesamt nahmen 429 Patienten an der Studie teil, von denen 42 aufgrund einer unklaren Melanomdiagnose ausgeschlossen wurden. Die verbleibenden 387 Teilnehmenden hatten ein Durchschnittsalter von M* = *54,12 Jahren (SD = 13,39). Es nahmen Melanompatient:innen aller Stadien (I–IV) teil; 28,7 % hatten lokoregionäre Metastasen (Stadium III) und 33,6 % litten an einer Fernmetastasierung (Stadium IV). Innerhalb dieser Stichprobe waren 57,9 % aktuell tumorfrei. Weitere soziodemografische und medizinische Merkmale der Stichprobe sind Tabelle 1 zu entnehmen.

**TABELLE 1:** Soziodemografische und medizinische Merkmale zum Zeitpunkt der Befragung.

	Mittelwert	Standardabweichung
***Alter* ^a^**	54,12	13,39
	n^b^	%^b^
***Geschlecht^c^***	212	54,80
Familienstand		
Ledig	57	14,70
Verheiratet	243	62,80
In einer Partnerschaft	37	9,60
Geschieden/getrennt	34	8,80
Verwitwet	16	4,10
***Kinder^d^***	267	69,00
***Bildungsstand***		
Universitätsabschluss	80	20,70
Hochschulreife	121	31,30
Mittlere Reife	95	24,50
Hauptschulabschluss	85	22,00
Kein Abschluss	6	1,60
***Berufliche Situation***		
Teilzeitbeschäftigt	60	15,50
Vollzeitbeschäftigt	134	34,60
In Ausbildung	3	0,80
Im Ruhestand	116	30,00
Hausfrau/‐mann	4	1,00
Nicht erwerbstätig	13	3,40
Krankgeschrieben	38	9,80
Sonstiges	19	4,90
***Tumorstadium***		
Stadium I	85	22,00
Stadium II	61	15,80
Stadium III	111	28,70
Stadium IV	130	33,60
***Behandlungssituation^e^***		
Tumorfrei	224	57,90
Nichtresektable Erkrankung/Metastasen nachweisbar	62	16,00
Konnte nicht beurteilt werden	101	26,10
***Aktuelle Behandlungsart^f^***		
Keine/Nachsorge	133	30,80
Operation	73	16,90
Immuntherapie	173	40,00
Zielgerichtete Therapie	31	7,20
Strahlentherapie	7	1,60
Andere Therapien	15	3,50

^a^ Mittelwert und Standardabweichung angegeben

^b^ n = Anzahl der Teilnehmenden, % = Anteil an der Gesamtstichprobe

^c^ Bezieht sich auf das weibliche Geschlecht

^d^ Bezieht sich auf Personen mit Kindern

^e^ die Patienten berichteten ihren aktuellen Behandlungsstatus selbst mit den Begriffen „kurativ“ oder „palliativ“, die Tabelle 1 zeigt die entsprechende medizinische Terminologie, wie sie von behandelnden Dermatolog:innen bereitgestellt wurde

^f^ mehr als eine Behandlungsart war möglich.

### Testkonstruktion

Zunächst werden einfache deskriptive Statistiken auf Itemebene berichtet. Anschließend folgen eine explorative Faktorenanalyse sowie die Überprüfung der Reliabilität. Zur Einschätzung der konvergenten und diskriminanten Validität wurden Zusammenhänge mit verwandten und nicht‐verwandten Konstrukten untersucht.

#### Deskriptive Grundauswertung und psychometrische Analyse

Es wurden Zusammenfassungsstatistiken der finalen Version des EMQoLI mit allen 33 Items berechnet. Die meisten Items zeigen eine deutlich rechtsschiefe Verteilung, was in der psychologischen Symptomerfassung,^38^ und Lebensqualitätsmessung,^39^ nicht unüblich ist. Dies deutet darauf hin, dass das EMQoLI eher eine beeinträchtigungsbezogene Variable erfasst als ein stabiles Persönlichkeitsmerkmal. Die relativ geringe Varianz symptombezogener Items – insbesondere zu Übelkeit, Aufstoßen, Durchfall und Fieber – weist auf eine niedrige Prävalenz dieser Symptome in der vorliegenden Stichprobe hin. Daher wurden polychorische Korrelationen für die Faktorenanalyse (EFA) verwendet.^40^

#### Itemauswahl und faktorenanalytische Validierung

Im ersten Validierungsschritt wurden die Items extrahiert, die besonders aussagekräftig für die Melanom‐spezifische Lebensqualität (und ihre Subdimensionen) sind. Um die optimale Itemanzahl und faktorielle Stabilität zu prüfen, wurde eine erste explorative Faktorenanalyse (EFA) durchgeführt, basierend auf der von verschiedenen Verfahren vorgeschlagenen Anzahl an Faktoren, wie sie im Befehl *n_factors* des *parameters*‐Pakets implementiert sind.^35^ Alle Items zeigten Kaiser‐Meyer‐Olkin‐Werte über 0,6, die meisten sogar über 0,9, was auf eine gute Eignung für Faktorenanalysen hinweist. Vier von 19 Verfahren (21,05 %) empfahlen eine Drei‐Faktoren‐Lösung. Acht Items mussten wegen Mehrfachladungen und niedriger Kommunalitäten ausgeschlossen werden (Tabelle S1 im Online‐Supplement), was auf inhaltliche Unklarheit oder Redundanz hindeutet. Aufgrund der hohen Relevanz, aber geringen Spezifität von Fatigue‐Symptomen wurde Item 3 („Ich litt unter Müdigkeit“) trotz Mehrfachladung in dem Faktor mit der höchsten Ladung beibehalten. Item 2 („Ich habe Schwellungen im Bereich der postoperativen Narbe des Melanoms und/oder der Metastasen“) wurde trotz einer niedrigen Kommunalität von 0,235 beibehalten, da es funktional bedeutsam für die allgemeine Lebensqualität ist.

Die verbleibenden 25 Items wurden erneut hinsichtlich der optimalen Faktorenzahl analysiert. Sieben von 19 Verfahren (36,8 %) unterstützten erneut eine Drei‐Faktoren‐Lösung. Eine anschließende Faktorenanalyse mit Varimax‐Rotation zeigte, dass alle Items Faktorladungen über 0,4 und Kommunalitäten über 0,25 aufwiesen (Tabelle S2 im Online‐Supplement). Dies spricht dafür, dass jedes Item eindeutig einer Subskala zugeordnet werden kann und kein Item redundant erscheint. Lediglich Item 3 („Ich leide unter Müdigkeit“) zeigt relevante Mehrfachladungen, die jedoch aufgrund der inhaltlichen Breite des Symptoms als akzeptabel bewertet wurden. Die durch die Faktorstruktur erklärte Varianz beträgt 49 %.

Die finale Version des EMQoLI besteht aus 25 Items, die auf einer fünfstufigen Likert‐Skala beantwortet werden. Sechs Items sind invertiert formuliert. Basierend auf dem Iteminhalt wurden die drei Subskalen des EMQoLI als *Symptome, Belastung und Ressourcen* interpretiert. Höhere EMQoLI‐Werte weisen auf eine stärkere Beeinträchtigung der gesundheitsbezogenen Lebensqualität (HRQoL) hin.

### Reliabilitätsanalysen

Auch die Reliabilitätskennwerte deuten auf ein zufriedenstellendes Maß an Zuverlässigkeit hin (siehe ergänzendes Material im Online‐Supplement). Alle drei Subskalen zeigen niedrige bis moderate Korrelationen miteinander: (Belastung x Symptome rho = 0,45, p < 0,001; Belastung x Ressourcen rho = 0,26, p < 0,001; Ressourcen x Symptome rho = 0,22, p < 0,001).

### Validität der EMQoLI‐Skala

*Zusammenhänge mit soziodemografischen und gesundheitsbezogenen Merkmalen*: Weibliche Teilnehmende zeigen erhöhte Werte auf allen Subskalen des EMQoLI sowie im Gesamtscore (alle p < 0,05), wobei der Unterschied auf der Subskala Belastung am deutlichsten ausfällt (Mann‐Whitney‐U‐Test: W = 24.880, p < 0,001). Zudem zeigt eine Spearman‐Korrelationsanalyse zwischen dem Alter der Teilnehmenden und den EMQoLI‐Werten, dass die wahrgenommene Belastung mit steigendem Alter abnimmt (ρ = –0,242, p < 0,001). Dieser Effekt trägt vermutlich auch zur signifikanten Korrelation zwischen Alter und Gesamtscore bei (ρ = –0,219, p < 0,001). Weitere Details zu Zusammenhängen mit anderen soziodemografischen und gesundheitsbezogenen Merkmalen sind dem ergänzenden Material zu entnehmen.

**ABBILDUNG 1:**
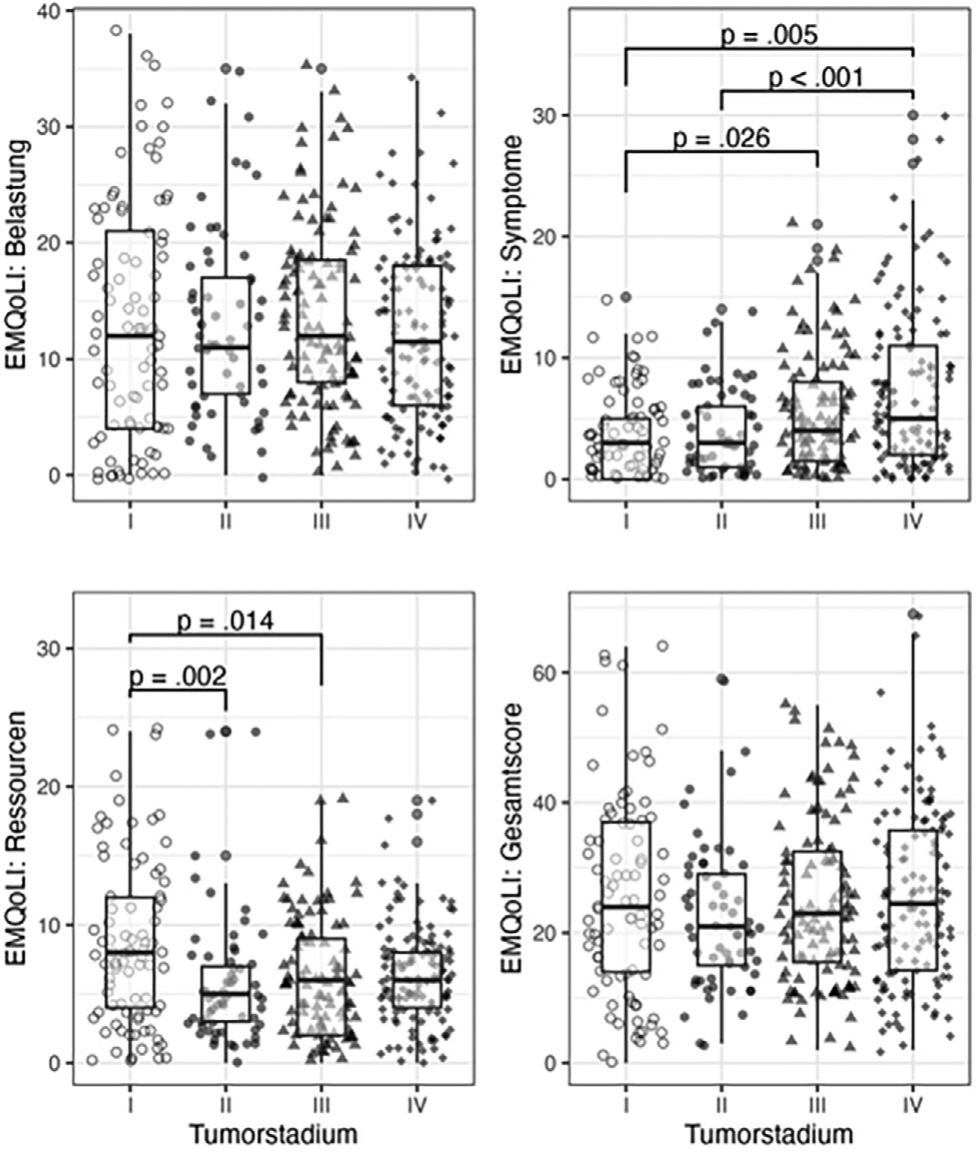
Unterschiede der EMQoLI‐Scores über die vier Tumorstadien. Signifikante Unterschiede zeigen sich hinsichtlich der Anzahl und Schwere der Symptome (oberes rechtes Feld) sowie der wahrgenommenen Ressourcen.

Unterschiede zwischen Tumorstadien: Zur Prüfung, ob die wahrgenommene gesundheitsbezogene Lebensqualität (HRQoL) mit dem Krankheitsstadium zusammenhängt, wurden die EMQoLI‐Werte zwischen Patienten der Tumorstadien I bis IV mittels Kruskal‐Wallis‐Tests verglichen (aufgrund schiefer Verteilungen). Für den Gesamtscore (χ^2^(3) = 2,512, p = 0,47) sowie die Subskala Belastung (χ^2^(3) = 1,761, p = 0,62) konnten keine signifikanten Unterschiede festgestellt werden. Signifikante Unterschiede ergaben sich jedoch für die Subskala Symptome (χ^2^(3) = 22,978, p < 0,001). Holm‐korrigierte Post‐hoc‐Dunn‐Tests zeigten Unterschiede zwischen: Tumorstadien I und III (z = 2,72, p = 0,026), Tumorstadien I und IV (z = 4,33, p < 0,001), sowie für Tumorstadien II und IV (z = 3,32, p < 0,001). Auch für die Subskala Ressourcen wurden signifikante Unterschiede festgestellt (χ^2^(3) = 14,97, p = 0,002). Hier zeigen die entsprechenden Post‐hoc‐Tests signifikante Unterschiede zwischen den Tumorstadien I und II (z = –3,57, p = 0,002) sowie zwischen Stadium I und III (z = –2,98, p = 0,014). Diese Gruppenunterschiede sind in Abbildung 1 dargestellt.

#### Konvergente und diskriminante Validität

Zur weiteren Validierung des EMQoLI wurden zusätzliche psychometrische Verfahren eingesetzt, um zu untersuchen, wie stark der EMQoLI mit anderen Messinstrumenten korreliert, die entweder thematisch ähnlich sind (zum Beispiel andere HRQoL‐Maße oder konstruktverwandte Maße wie Depressions‐ und Angstsymptome), oder mit dem Konzept eher nicht verwandt sind (zum Beispiel Impulsivität).

#### Generische Maße der gesundheitsbezogenen Lebensqualität (HRQoL)

**ABBILDUNG 2:**
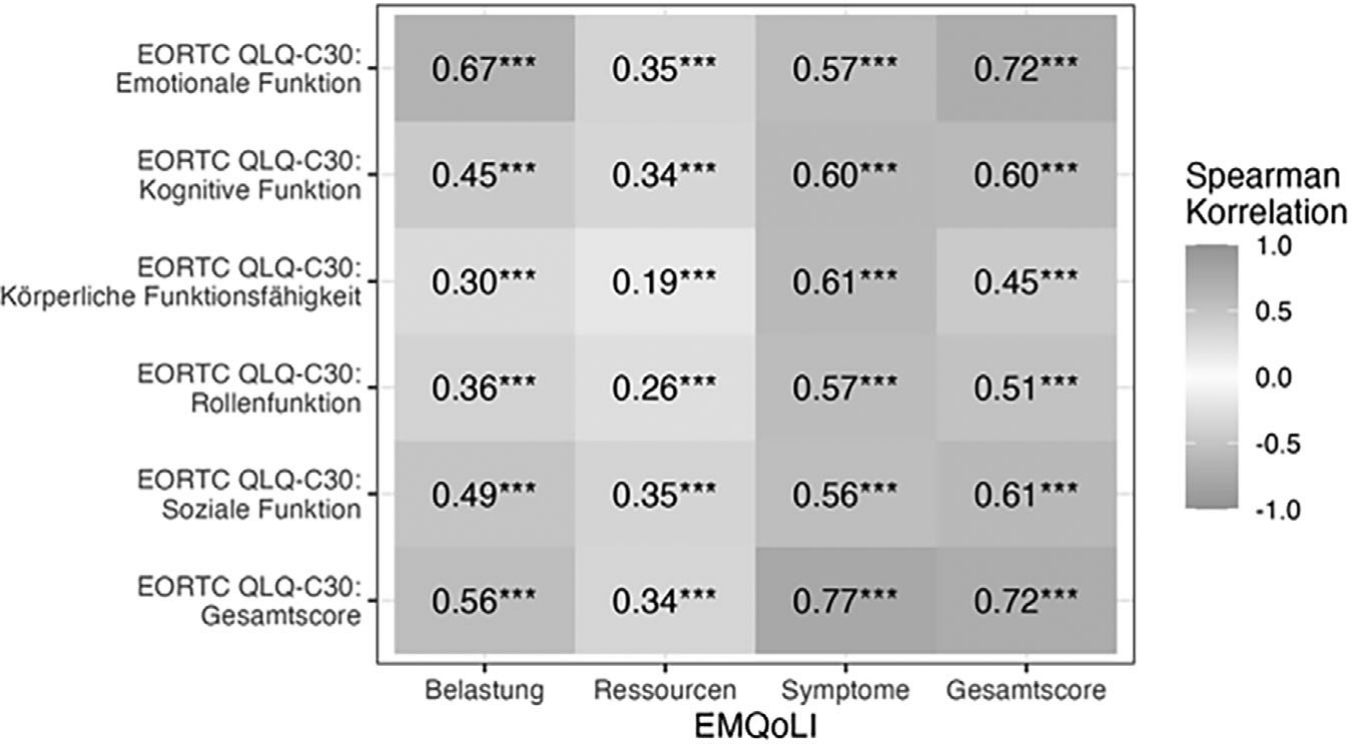
Holm‐korrigierte Korrelationen zwischen den EMQoLI‐ und den EORTC‐QLQ‐C30‐Skalen.

Es wurden Spearman‐Korrelationskoeffizienten zwischen jeder Subskala sowie dem Gesamtscore des EMQoLI und dem EORTC QLQ‐C30 berechnet. Die Ergebnisse sind im Korrelationsdiagramm in Abbildung 2 dargestellt. Alle Koeffizienten sind statistisch signifikant und deuten darauf hin, dass höhere EMQoLI‐Werte mit höheren EORTC QLQ‐C30‐Werten einhergehen. Dabei zeigt die Subskala *Ressourcen* des EMQoLI die geringsten Korrelationen mit den Skalen des EORTC QLQ‐C30.

#### Indikatoren psychischer Gesundheit

Es wurden ebenfalls Spearman‐Korrelationen berechnet, um den Zusammenhang zwischen EMQoLI (und seinen Subskalen) sowie den Angst‐ und Depressionswerten, als auch der allgemeinen Selbstwirksamkeit – einem wichtigen Schutzfaktor im Bereich psychischer Gesundheit – zu untersuchen.

**ABBILDUNG 3:**
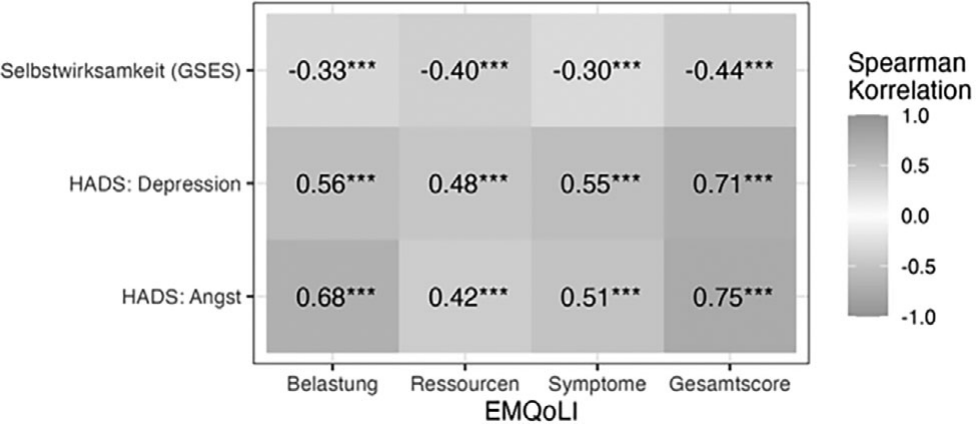
Holm‐korrigierte Korrelationen zwischen den EMQoLI‐Scores und Markern der psychischen Gesundheit.

Die Korrelationen zwischen EMQoLI und der Selbstwirksamkeit sind durchweg negativ (Abbildung 3), während die EMQoLI‐Werte positiv mit Angst und Depression korrelieren.

**ABBILDUNG 4:**
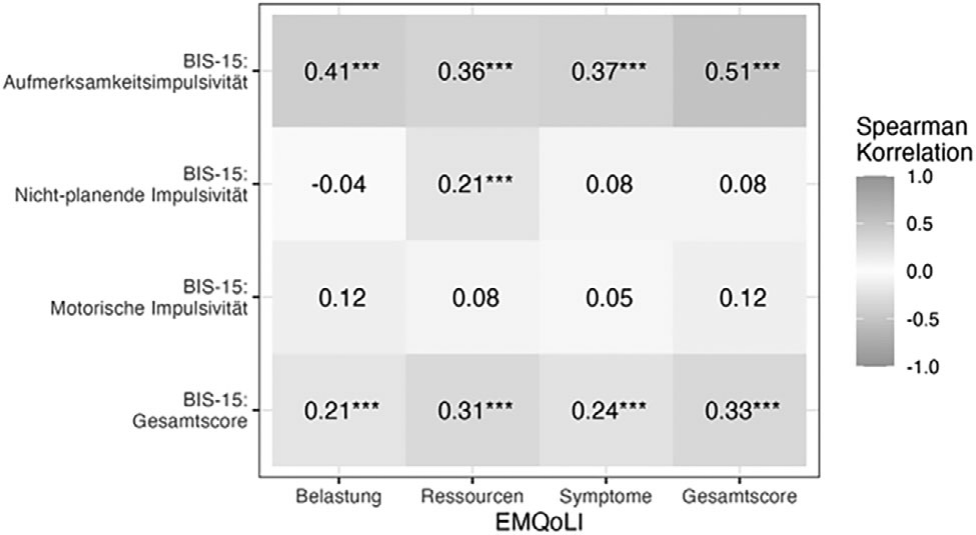
Holm‐korrigierte Korrelationen zwischen den EMQoLI‐Scores und der BIS‐15.

Zur Überprüfung der diskriminanten Validität wurden die Korrelationen zwischen dem EMQoLI und Impulsivität, gemessen mittels der BIS‐15, analysiert (Abbildung 4). Signifikante Zusammenhänge zeigten sich vor allem zwischen EMQoLI und der Aufmerksamkeitsimpulsivität.

## DISKUSSION

Das Ausbleiben somatischer Beschwerden nach einer Melanomtherapie bedeutet nicht automatisch, dass eine gute gesundheitsbezogene Lebensqualität vorliegt. Eine ganzheitliche Betrachtung der Melanomtherapie und der HRQoL‐Erfassung sollte neben körperlichen Symptomen wie Schmerzen auch psychologische Aspekte – etwa die Angst vor einem Rückfall – sowie psychosoziale Faktoren, wie soziale Unterstützung berücksichtigen.^7^, ^11^, ^28^, ^41^ Ziel der vorliegenden Studie war es daher, ein Instrument zur Erfassung der HRQoL bei Melanompatient:innen zu entwickeln und zu validieren – unabhängig vom Tumorstadium –, das sowohl therapiebedingte Nebenwirkungen neuer Behandlungsformen als auch psychische Belastungen, psychosoziale Aspekte, Unsicherheit und Sorgen erfasst.

An der Entwicklung und Validierung des EMQoLI nahm eine große Stichprobe deutschsprachiger Melanompatient:innen teil. Eine explorative Faktorenanalyse zeigte eine insgesamt gute Passung für ein Drei‐Faktoren‐Modell mit 25 Items, die den Subskalen *Belastung, Ressourcen* und *Symptome* zugeordnet wurden (Tabelle S2 im Online‐Supplement). Das EMQoLI und seine Subskalen zeigten in den Reliabilitätsanalysen eine zufriedenstellende interne Konsistenz. Darüber hinaus belegten Korrelationsanalysen eine gute konvergente sowie ausreichende diskriminante Validität: Signifikante Zusammenhänge zwischen EMQoLI, EORTC QLQ‐C30 und HADS‐Skalen für Angst und Depression bestätigten die konvergente Validität. Ebenso zeigte sich eine negative Korrelation mit der deutschen Selbstwirksamkeitsskala (GSE), was nahelegt, dass eine höhere Belastung durch das Melanom mit einer niedrigeren wahrgenommenen Selbstwirksamkeit einhergeht. Die diskriminante Validität wurde durch geringe Korrelationen mit den Subskalen für motorische und nicht‐planende Impulsivität der BIS‐15 belegt. Wichtig ist in diesem Zusammenhang, dass die BIS‐15‐Subskala „Aufmerksamkeitsimpulsivität“ Items enthält, die Symptome von Angst und Unruhe erfassen – ähnlich wie die HADS. Aufgrund dieser inhaltlichen Überlappung ist die beobachtete positive Korrelation zwischen EMQoLI und dieser Subskala nicht als mangelnde diskriminante Validität zu interpretieren. Diese Skala ist daher nicht ideal geeignet, um die diskriminante Validität des EMQoLI zu prüfen. Da es sich bei der vorliegenden Studie um die erste Evaluation des EMQoLI handelt, ist weitere psychometrische Forschung erforderlich.

In dieser Studie, die Teilnehmer aus allen vier Tumorstadien einschloss, zeigten weibliche Teilnehmende signifikant höhere EMQoLI‐Werte, was auf eine niedrigere HRQoL hinweist. Dieser Effekt war besonders ausgeprägt auf der Subskala *Belastung*. Dieses Ergebnis steht im Einklang mit früheren Untersuchungen zu geschlechtsspezifischen Unterschieden in der Krankheitslast bei Melanompatient:innen.^27^, ^42^ Darüber hinaus zeigte sich eine signifikante negative Korrelation zwischen dem *Alter* und der *Belastung*, was ebenfalls mit bestehender Forschung übereinstimmt.^43^ Höhere Tumorstadien waren mit einer signifikant stärkeren körperlichen Belastung auf der Subskala *Symptome* assoziiert, was frühere Erkenntnisse stützt, die einen Zusammenhang zwischen Tumorstadium und somatischen Beschwerden zeigen.^9^ Auf der Subskala *Ressourcen* berichteten Patienten im Stadium I von einem geringeren Gefühl der Unterstützung durch ihr privates Umfeld und medizinisches Personal sowie von weniger Zuversicht in Bezug auf die Zukunft im Vergleich zu Patienten in Stadium II und III. Dieses Ergebnis ist konsistent mit Studien, die darauf hinweisen, dass Patienten in niedrigeren Stadien aufgrund der vergleichsweise guten Prognose weniger psychosoziale Aufmerksamkeit erhalten.^26^ Allerdings zeigten sich auf der Ressourcenskala keine konsistenten oder linearen Zusammenhänge mit dem Tumorstadium. Die Überlappung zwischen den Gruppen, wie in den Boxplots sichtbar, deutet auf eine hohe individuelle Variabilität hin.^44^ Unabhängig vom Tumorstadium ist das EMQoLI jedoch in der Lage, Personen mit hoher körperlicher und psychischer Belastung und begrenzten Ressourcen zu identifizieren, was seine Anwendbarkeit über alle Stadien hinweg unterstreicht.

Mehrere Einschränkungen sind bei der Interpretation der vorliegenden Ergebnisse zu berücksichtigen. Die Verteilung der Teilnehmenden nach Melanomstadien ist in der untersuchten Stichprobe nicht gleichmäßig (Tabelle 1): Ein höherer Anteil entfällt auf Patienten mit Stadium III (28,7  %) und Stadium IV (33,6  %) im Vergleich zu Stadium I (22,0  %) und Stadium II (15,8  %). Zum Vergleich: In Deutschland haben etwa 75  % der Melanompatient:innen ein Melanom im Stadium I.^45^ Zudem weist die vorliegende Stichprobe ein geringeres Durchschnittsalter auf als die allgemeine Melanompopulation in Deutschland. Die Studienteilnahme erfolgte freiwillig, und ein Großteil der Teilnehmenden wurde über ein tertiäres Referenzzentrum für Hautkrebserkrankungen rekrutiert. Dadurch können Selektions‐ und Selbstselektionsverzerrungen nicht ausgeschlossen werden. Ein weiterer Punkt ist, dass die Datenerhebung während der COVID‐19‐Pandemie stattfand, was zu einer Verlängerung des Erhebungszeitraums auf rund 3 Jahre führte. Auch wenn aktuelle Forschungsergebnisse darauf hinweisen, dass die Pandemie keinen signifikanten Einfluss auf das Belastungs‐ oder Angstniveau von Krebspatienten im Vergleich zu gesunden Kontrollpersonen hatte,^46^ sollte der Zeitpunkt der Befragung und der pandemische Kontext bei der Interpretation der Ergebnisse berücksichtigt werden. Darüber hinaus basiert die Studie ausschließlich auf Selbstauskünften, was die objektive Überprüfung von Melanomdiagnose, Tumorstadium und Behandlung einschränkt. In Zweifelsfällen konnten Teilnehmenden aus tertiären Referenzzentren ihre Angaben jedoch durch ihre lokale dermatoonkologische Betreuungsperson bestätigen lassen.

Schließlich wurde der Fragebogen bislang nur auf Deutsch validiert und ist daher nicht ohne Weiteres in anderen Sprachen einsetzbar. Eine englischsprachige Validierung befindet sich derzeit in Arbeit und wird in Kürze für Fachkräfte und Patienten im englischsprachigen Raum zur Verfügung stehen.

Zusammenfassend lässt sich festhalten, dass die Therapie des Melanoms in den vergangenen Jahren erhebliche Fortschritte gemacht hat, was jedoch zugleich mit einem erhöhten Potenzial für therapiebedingte Nebenwirkungen einhergeht. Die Fachliteratur wie auch die vorliegende Studie zeigen, dass bei Melanompatienten ein erheblicher Bedarf an psychosozialer Unterstützung besteht und dass sowohl die Erkrankung selbst als auch ihre Behandlung mit psychischer Belastung einhergehen können. Die frühe Erkennung psychischer und somatischer Belastungen sowie des Bedarfs an psychosozialer Unterstützung kann sich positiv auf die Versorgungsqualität auswirken und langfristig die Lebensqualität und Genesung verbessern. Da ein entsprechendes Instrument bislang fehlte, wurde das EMQoLI entwickelt – ein neues Messinstrument, das diese Aspekte in ihrer Gesamtheit erfasst und somit die Versorgung von Melanompatient:innen verbessert. Das EMQoLI wurde gezielt entwickelt, um die Schwächen bestehender Instrumente zu überwinden. Es misst die Melanom‐spezifische gesundheitsbezogene Lebensqualität und berücksichtigt dabei insbesondere auch therapiebedingte Nebenwirkungen von Immun‐ und zielgerichteter Therapien. Das EMQoLI ist zuverlässig, validiert auf Deutsch, und für Patienten aller Tumorstadien anwendbar. Es handelt sich um ein Inventar bestehend aus einem kurzen und benutzerfreundlichen Fragebogen, der Fachpersonen im Gesundheitswesen hilft, Patienten mit einem erhöhten Bedarf an psychosozialer Unterstützung frühzeitig zu identifizieren. Durch den Einsatz des EMQoLI kann nicht nur festgestellt werden, ob ein Unterstützungsbedarf besteht, sondern auch in welchen Bereichen – Symptome, Ressourcen oder Belastung – Patienten besonders betroffen sind. Dadurch lassen sich geeignete Interventionen und Unterstützungsangebote gezielter und effizienter gestalten. Insgesamt leistet das EMQoLI somit einen bedeutenden Beitrag zur psychoonkologischen Versorgung von Menschen mit Melanom.

## DANKSAGUNG

Open access Veröffentlichung ermöglicht und organisiert durch Projekt DEAL.

## INTERESSENKONFLIKT

Die Autoren geben folgende finanzielle Interessen/persönliche Beziehungen an, die als potenzielle Interessenkonflikte angesehen werden können:

**GL** berichtet über eine beratende Funktion oder hat Honorare/Reisekosten von Novartis, Pierre Fabre und Sunpharma erhalten.

**LZ** war beratend tätig und hat Honorare von BMS, MSD, Novartis, Pierre Fabre, Regeneron und Sunpharma sowie Reisekostenzuschüsse von MSD, BMS, Pierre Fabre, Regeneron, Sunpharma und Novartis außerhalb der eingereichten Arbeit erhalten.

**DS** berichtet über Zuschüsse oder Verträge von BMS, Amgen, MSD und Novartis, alle an die Einrichtung. Beratungshonorare von BMS, Novartis, MSD, Moderna, BioNTech, Regeneron, Pierre Fabre, Pfizer, Boehringer Ingelheim, Replimune, Sunpharma, Philogen, Skyline Dx, Daiichi Sanyo, Ipsen, IoVance, IOBioTech, Immunocore, BioAlta, Immatics, Seagen und Merck‐Serono. Zahlungen oder Honorare für Vorträge, Präsentationen, Referententätigkeiten, das Verfassen von Manuskripten oder Bildungsveranstaltungen von BMS, Novartis, MSD, Pierre Fabre, Regeneron, Sunpharma und Regeneron. Unterstützung für die Teilnahme an Tagungen und/oder Reisen von Pierre Fabre. Teilnahme an einem Datensicherheitssgremium oder Beirat von BMS, Novartis, MSD, Moderna, BioNTech, Regeneron, Pierre Fabre, Replimune, Philogen, Skyline Dx, Daiichi Sanyo, IMCheck, Ipsen, IoVance, IOBioTech, Immunocore, BioAlta und Immatics. Führungs‐ oder treuhänderische Rolle in anderen Gremien, Gesellschaften, Ausschüssen oder Interessenvertretungen, bezahlt oder unbezahlt: Dermatologic Cooperative Group (DeCOG, unbezahlt), EuMelaREg (unbezahlt), NVKH (unbezahlt) und Deutsche Hautkrebsstiftung/Hiege‐Stiftung (unbezahlt).

**EMS** berichtet über Sponsoring‐ und Ad‐Board‐Aktivitäten für Novartis Pharma GmbH.

**EL** berichtet über eine beratende Funktion oder hat Honorare/Reisekosten von Bristol‐Myers Squibb, Merck Sharp & Dohme, Kyowa Kirin, Novartis, Pierre Fabre, Regeneron, Sanofi, Sunpharma und Takeda erhalten.

**MT** berichtet über Sponsoring‐ und Ad‐Board‐Aktivitäten für Novartis Pharma GmbH.

Sofern weitere Autoren vorhanden sind, erklären diese, dass ihnen keine konkurrierenden finanziellen Interessen oder persönlichen Beziehungen bekannt sind.

## Supporting information



Supplementary information
