# Supplementary material for: Entwicklung und Validierung des Essen Melanoma Quality of Life Inventory (EMQoLI)
Source: J Dtsch Dermatol Ges. 2026 Sep 8;24(9):1241–50. [Article in German] doi: 10.1111/ddg.15998_g (PMC13551206; doi:10.1111/ddg.15998_g)
Supplement: Supplementary file 1 [file DDG-24-1241-s001.docx]

Ergänzendes Online-Material („supplemental online material“)

zu

Entwicklung und Validierung des Essen Melanoma Quality of Life Inventory (EMQoLI)

Musche V., Schadendorf T., Schweda A., Lodde G., Neumann J., Zimmer L., Hüning S., Schadendorf D., Skoda E.-M., Livingstone E. & Teufel M.

**Ergänzende Ergebnisse**

**Explorative Faktorenanalysen und Itemauswahl**

Im Hauptmanskript beschreiben wir, dass eine erste Faktorenanalyse mit Varimax-Rotation unter Einbeziehung aller 33 Items zu einer eher suboptimalen Lösung mit Kreuzladungen („cross loadings“) über eine Reihe von Items sowie niedrigen Kommunalitäten („communalities“) führte. Die ergänzende Tabelle S1 (siehe unten) fasst die Ergebnisse einer solchen Lösung zusammen. Es ist anzumerken, dass wir den Items 2 und 3 („Ich habe Schwellungen im Bereich der postoperativen Narbe des Melanoms und/oder der Metastasen“ und „Ich leide unter Müdigkeit“) aufgrund ihrer hohen theoretischen Relevanz und ihrer zentralen Bedeutung für die Lebensqualität von Patientinnen und Patienten mit malignen Melanomen besondere Bedeutung beigemessen haben. Sie wurden daher unabhängig von ihren Faktorladungen und Kommunalitäten im Fragebogen beibehalten. Auf Basis der ersten explorativen Faktorenanalyse wurden zuletzt also insgesamt acht Items ausgeschlossen; die entsprechenden Ergebnisse sind in der ergänzenden Tabelle S2 dargestellt. Hier haben wir auch die Item-Kommunalitäten sowie die Item-Skala- und Item-Gesamt-(Spearman-)Korrelationen aufgeführt, die zufriedenstellende Werte aufweisen.

**Reliabilitätsanalysen**

Die Reliabilitätsindizes weisen ebenfalls auf eine zufriedenstellende Reliabilität hin, wobei MacDonalds Ωtotal – das auf der Grundlage derselben Faktorstruktur wie oben berechnet wurde – hier Werte von 0,93 und ein Cronbachs α von 0,90 ergibt. Alle drei Subskalen zeigen moderate bis mäßige Korrelationen mit anderen Subskalen (Belastung x Symptome rho = 0,45, p < 0,001; Belastung x Ressourcen rho = 0,26, p < 0,001; Ressourcen x Symptome rho = 0,22, p < 0,001).

**Zusammenhang zwischen den EMQoLI-Subskalen und soziodemografischen Daten**

Zusätzlich zur Untersuchung der Unterschiede in den EMQOLI-Werten für verschiedene Krebsstadien prüften wir auch Zusammenhänge mit soziodemografischen Variablen. Hier werden weitere Zusammenhänge mit Bildung, Familienstand und Beschäftigungsstatus beobachtet. Ein Kruskal-Wallis-Test zur Untersuchung von Unterschieden in den EMQoLI-Werten zwischen verschiedenen Bildungsniveaus zeigte signifikante Gruppenunterschiede im EMQoLI-Gesamtwert (χ²(4) = 11.16, p = .025). Nachfolgende Dunn-Tests ergaben jedoch lediglich auf unkorrigiertem Niveau (d. h. ohne Holm-Korrektur, p = .022) eine höhere Beeinträchtigung der gesundheitsbezogenen Lebensqualität bei Personen mit Hochschulabschluss im Vergleich zu Personen mit Abitur. Für den Familienstand konnten keine Auswirkungen festgestellt werden (alle p > 0,05). Was den Beschäftigungsstatus der Teilnehmer betrifft, so weisen Personen, die angeben, noch in Teilzeit zu arbeiten, höhere EMQoLI-Gesamtwerte auf als Rentner (χ^2^ (4) = 28,418, p < 0,001; Holm-korrigierter Dunn-Test Z = -3,362, p = 0,028). Vollzeitbeschäftigte und Rentner weisen eine geringere HRQoL-Belastung auf als Personen im Krankenstand (Z = 3,309, p = 0,025; Z = 4,268, p < 0,001; Holm-korrigiert). Diese Muster in der Gesamtpunktzahl scheinen auf Unterschiede in den Subskalen „Symptome” und „Belastung” zurückzuführen zu sein, wobei Personen im Krankenstand im Allgemeinen eine höhere Belastung aufweisen. Zudem zeigten die Mann-Whitney-U-Tests für alle EMQoLI-Skalen eine höhere Gesamtbelastung bei Personen mit diagnostizierten psychischen Erkrankungen (alle p < .001).

**Analyse der Pilotdaten**

Vor der vollständigen Datenerhebung wurde eine Pilotstudie mit einer separaten Stichprobe von 59 Teilnehmenden durchgeführt, um erste Hinweise auf die Reliabilität und Validität des EMQoLI zu gewinnen. Diese Stichprobe ist unabhängig und nicht in die im vorliegenden Manuskript berichteten Hauptanalysen einbezogen.

In diesem Rahmen führten wir eine erste Überprüfung der Itemverteilungen durch und bewerteten die Korrelationen zwischen den Items sowie die Assoziationen mit externen Kriterien, um die vorläufige Kriteriumsvalidität zu beurteilen. Die meisten Items wiesen eine ausreichende Varianz auf (siehe ergänzende Abbildung S1 und ergänzende Tabelle S3), obwohl einige symptombezogene Items ebenso wie in der endgültigen Validierungsstichprobe eine geringere Variabilität aufwiesen. Aufgrund ihrer Relevanz für die Behandlung von Melanomen wurden diese Items im Instrument beibehalten. Wir analysierten zudem das Gesamtmuster der Item-Korrelationen, das sich überwiegend positiv zeigte (siehe ergänzende Abbildung S2 für die Spearman-Korrelationen), und fanden eine insgesamt zufriedenstellende interne Konsistenz (Cronbachs α = .90). Aufgrund der kleinen Stichprobe führten wir keine Faktorenanalysen durch. Um erste Hinweise auf die Kriteriumsvalidität zu erhalten, untersuchten wir die Zusammenhänge zwischen dem EMQoLI-Gesamtscore und den Scores der zur Validierung genutzten etablierten Instrumente zur Erfassung von Impulsivität, gesundheitsbezogener Lebensqualität (EORTC QLQ-C30), Selbstwirksamkeit, Depression und Angst (siehe ergänzende Abbildung S3). Die Ergebnisse dieser Pilotstudie lieferten ausreichende Evidenz, um mit der Hauptdatenerhebung fortzufahren.

**Vorläufige englische Version des Essen Melanoma Quality of Life Inventory (EMQoLI) nur zu Übersichtszwecken**

1. Pain at the surgery site
2. Swelling at the surgery site
3. Tiredness
4. Joint pain
5. Loss of appetite
6. Rash or itching
7. Nausea
8. Vomiting
9. Diarrhea
10. Fever
11. Other adverse events
12. Feeling supported by doctors
13. Fear of cancer returning/ condition worsening
14. Hopeful about the future
15. Satisfied with provided infomation by doctor‘s
16. Others did not understand my illness
17. Fear of follow-up results
18. Worried about outdoor activities in the sun
19. Supported by the healthcare system
20. Worried about family’s melanoma risk
21. Fear of dying
22. Bothered by surgery scar
23. Understood treatment options
24. Pressure to check skin
25. Supported by family and friends

Ergänzende Tabelle S1

|  |  | Faktor 1: Belastung | Faktor 2: Symptomskala | Faktor 3: Ressourcen | Kommunalität | Ausschluss |
| --- | --- | --- | --- | --- | --- | --- |
| 1 | Schmerzen an der Operationsseite | 0.53 | 0.10 | 0.07 | 0.295 |  |
| 2 | Schwellungen an der Operationsstelle | 0.46 | 0.12 | 0.08 | 0.235 |  |
| 3 | Müdigkeit | 0.41 | 0.58 | 0.15 | 0.526 |  |
| 4 | Gelenkschmerzen | 0.24 | 0.54 | 0.23 | 0.398 |  |
| 5 | Appetitlosigkeit | 0.12 | 0.67 | -0.04 | 0.464 |  |
| 6 | Hautausschlag oder Juckreiz | 0.27 | 0.45 | 0.03 | 0.280 |  |
| 7 | Übelkeit | 0.16 | 0.84 | 0.03 | 0.736 |  |
| 8 | Erbrechen | 0.01 | 0.90 | -0.03 | 0.806 |  |
| 9 | Durchfall | 0.16 | 0.70 | 0.08 | 0.524 |  |
| 10 | Fieber | 0.08 | 0.77 | -0.04 | 0.594 |  |
| 11 | Sonstige unerwünschte Ereignisse | 0.21 | 0.67 | 0.02 | 0.496 |  |
| 12 | Besorgnis über anhaltende Nebenwirkungen | 0.41 | 0.50 | 0.09 | 0.422 | x |
| 13 | Gefühl der Unterstützung durch Ärzte | -0.01 | 0.01 | 0.72 | 0.521 |  |
| 14 | Depressive Verstimmung aufgrund des Melanoms | 0.65 | 0.44 | 0.14 | 0.644 | x |
| 15 | Angst vor einem Rückfall/einer Verschlechterung des Zustands | 0.82 | 0.23 | 0.01 | 0.724 |  |
| 16 | Einschränkungen aufgrund des Melanoms | 0.56 | 0.53 | 0.17 | 0.623 | x |
| 17 | Hoffnungsvoll in Bezug auf die Zukunft | 0.24 | 0.18 | 0.48 | 0.325 |  |
| 18 | Zufriedenheit mit den Informationen der Ärzte | 0.13 | 0.09 | 0.80 | 0.669 |  |
| 19 | Andere haben meine Krankheit nicht verstanden | 0.63 | 0.19 | 0.32 | 0.538 |  |
| 20 | Angst vor den Nachuntersuchungen | 0.76 | 0.23 | 0.01 | 0.635 |  |
| 21 | Sorgen aufgrund von Außenaktivitäten in der Sonne | 0.59 | 0.19 | 0.05 | 0.391 |  |
| 22 | Schuldgefühle, weil ich meinen Verpflichtungen nicht nachkommen kann | 0.58 | 0.46 | 0.24 | 0.604 | x |
| 23 | Unterstützung durch das Gesundheitssystem | 0.03 | -0.01 | 0.54 | 0.294 |  |
| 24 | Sorge um das Melanomrisiko von Verwandten | 0.55 | 0.13 | 0.02 | 0.317 |  |
| 25 | Unbehagen bei Intimität | 0.44 | 0.46 | 0.24 | 0.460 | x |
| 26 | Bedauern über frühere Sonnenexposition | 0.45 | 0.07 | -0.05 | 0.208 | x |
| 27 | Angst vor dem Tod | 0.69 | 0.26 | 0.04 | 0.543 |  |
| 28 | Sorge, dass die Bewältigung der täglichen Anforderungen nicht möglich ist | 0.55 | 0.56 | 0.14 | 0.639 | x |
| 29 | Störung durch Operationsnarbe | 0.60 | 0.00 | 0.16 | 0.386 |  |
| 30 | Verständnis der Behandlungsmöglichkeiten | 0.02 | 0.07 | 0.70 | 0.501 |  |
| 31 | Druck, die Haut zu überprüfen | 0.62 | 0.06 | 0.06 | 0.391 |  |
| 32 | Akzeptanz der Krankheit | 0.05 | -0.04 | 0.47 | 0.223 | x |
| 33 | Unterstützung durch Familie und Freunde | 0.10 | 0.10 | 0.69 | 0.497 |  |

Erstes Ergebnis einer explorativen Faktorenanalyse mit drei Faktoren unter Verwendung einer Varimax-Rotation aller 33 ursprünglichen Items. Die schattierten Bereiche zeigen Faktorladungen über .40 sowie Kommunalitäten unterhalb des Grenzwerts von .25.

Ergänzende Tabelle S2

|  |  |  | Faktor 1: Belastung |  | Faktor 2: Symptomskala |  | Faktor 3: Ressourcen |  | Kommunalität | Korrelation auf Item-Ebene | Korrelation zwischen Item und Gesamtwert |
| --- | --- | --- | --- | --- | --- | --- | --- | --- | --- | --- | --- |
| 1 | Schmerzen an der Operationsstelle |  | 0.57 |  | 0.11 |  | 0.07 |  | 0.344 | 0.527 | 0.441 |
| 2 | Schwellungen an der Operationsstelle |  | 0.49 |  | 0.12 |  | 0.06 |  | 0.263 | 0.459 | 0.392 |
| 3 | Müdigkeit |  | 0.40 |  | 0.56 |  | 0.17 |  | 0.499 | 0.801 | 0.623 |
| 4 | Gelenkschmerzen |  | 0.23 |  | 0.52 |  | 0.25 |  | 0.387 | 0.709 | 0.516 |
| 5 | Appetitlosigkeit |  | 0.13 |  | 0.68 |  | -0.02 |  | 0.484 | 0.554 | 0.351 |
| 6 | Hautausschlag oder Juckreiz |  | 0.28 |  | 0.45 |  | 0.04 |  | 0.281 | 0.559 | 0.413 |
| 7 | Übelkeit |  | 0.17 |  | 0.86 |  | 0.06 |  | 0.766 | 0.562 | 0.429 |
| 8 | Erbrechen |  | 0.01 |  | 0.90 |  | 0.00 |  | 0.808 | 0.334 | 0.257 |
| 9 | Durchfall |  | 0.19 |  | 0.73 |  | 0.11 |  | 0.583 | 0.498 | 0.376 |
| 10 | Fieber |  | 0.08 |  | 0.79 |  | -0.02 |  | 0.637 | 0.325 | 0.243 |
| 11 | Sonstige unerwünschte Ereignisse |  | 0.21 |  | 0.62 |  | 0.03 |  | 0.435 | 0.578 | 0.385 |
| 12 | Gefühl, von Ärzten unterstützt zu werden |  | 0.00 |  | -0.01 |  | 0.74 |  | 0.522 | 0.724 | 0.353 |
| 13 | Angst vor einem Rückfall/einer Verschlechterung des Zustands |  | 0.78 |  | 0.22 |  | 0.01 |  | 0.657 | 0.775 | 0.655 |
| 14 | Hoffnungsvoll in Bezug auf die Zukunft |  | 0.23 |  | 0.15 |  | 0.46 |  | 0.286 | 0.593 | 0.498 |
| 15 | Zufrieden mit den vom Arzt bereitgestellten Informationen |  | 0.13 |  | 0.06 |  | 0.86 |  | 0.757 | 0.750 | 0.471 |
| 16 | Andere haben meine Krankheit nicht verstanden |  | 0.64 |  | 0.17 |  | 0.32 |  | 0.540 | 0.603 | 0.612 |
| 17 | Angst vor den Ergebnissen der Nachuntersuchung |  | 0.78 |  | 0.23 |  | 0.02 |  | 0.669 | 0.787 | 0.680 |
| 18 | Sorgen aufgrund von Außenaktivitäten in der Sonne |  | 0.59 |  | 0.20 |  | 0.06 |  | 0.390 | 0.651 | 0.555 |
| 19 | Unterstützung durch das Gesundheitssystem |  | 0.04 |  | -0.03 |  | 0.55 |  | 0.304 | 0.633 | 0.311 |
| 20 | Sorge wegen des Melanomrisikos in der Familie |  | 0.54 |  | 0.12 |  | 0.02 |  | 0.303 | 0.588 | 0.455 |
| 21 | Angst vor dem Tod |  | 0.66 |  | 0.25 |  | 0.07 |  | 0.504 | 0.728 | 0.626 |
| 22 | Störung durch Operationsnarbe |  | 0.61 |  | -0.01 |  | 0.14 |  | 0.397 | 0.524 | 0.427 |
| 23 | Verständnis der Behandlungsmöglichkeiten |  | 0.03 |  | 0.05 |  | 0.67 |  | 0.456 | 0.678 | 0.367 |
| 24 | Druck, die Haut zu überprüfen |  | 0.63 |  | 0.08 |  | 0.06 |  | 0.411 | 0.643 | 0.513 |
| 25 | Unterstützung durch Familie und Freunde |  | 0.11 |  | 0.09 |  | 0.67 |  | 0.464 | 0.597 | 0.389 |
|  |  |  |  |  |  |  |  |  |  |  |  |

Die endgültige faktorielle Lösung auf Basis einer explorativen Faktorenanalyse mit Varimax-Rotation und drei Faktoren für die verbleibenden 25 Items ist in der ergänzenden Tabelle S1 dargestellt. Die schattierten Bereiche markieren erneut dominante Faktorladungen über .40. Sowohl die Kommunalitäten als auch die Item-Skalen- und Item-Gesamt-Korrelationen weisen zufriedenstellende Werte auf.

Ergänzende Tabelle S3

|  |  | M | SD |  | Median | 25. Perzentil | 75. Perzentil | Schiefe | Kurtosis |
| --- | --- | --- | --- | --- | --- | --- | --- | --- | --- |
| 1 | Schmerzen an der Operationsstelle | 0.75 | 0.92 |  | 0 | 0 | 1 | 0.95 | -0.19 |
| 2 | Schwellungen an der Operationsstelle | 0.61 | 1.03 |  | 0 | 0 | 1 | 1.92 | 3.17 |
| 3 | Müdigkeit | 1.25 | 1.03 |  | 1 | 1 | 2 | 0.65 | -0.26 |
| 4 | Gelenkschmerzen | 1.05 | 1.33 |  | 0 | 0 | 2 | 0.99 | -0.23 |
| 5 | Appetitlosigkeit | 0.24 | 0.60 |  | 0 | 0 | 0 | 2.39 | 4.33 |
| 6 | Hautausschlag oder Juckreiz | 0.81 | 1.21 |  | 0 | 0 | 1 | 1.52 | 1.35 |
| 7 | Übelkeit | 0.24 | 0.68 |  | 0 | 0 | 0 | 3.79 | 16.90 |
| 8 | Erbrechen | 0.14 | 0.60 |  | 0 | 0 | 0 | 5.39 | 31.57 |
| 9 | Durchfall | 0.47 | 1.02 |  | 0 | 0 | 0 | 2.42 | 5.45 |
| 10 | Fieber | 0.12 | 0.49 |  | 0 | 0 | 0 | 4.71 | 23.32 |
| 11 | Sonstige unerwünschte Nebenwirkungen | 0.34 | 0.84 |  | 0 | 0 | 0 | 2.85 | 8.08 |
| 12 | Besorgnis über anhaltende Nebenwirkungen | 0.85 | 1.16 |  | 0 | 0 | 1 | 1.35 | 1.02 |
| 13 | Gefühl der Unterstützung durch Ärzte | 0.88 | 1.20 |  | 0 | 0 | 1 | 1.28 | 0.59 |
| 14 | Depressive Verstimmung aufgrund des Melanoms | 1.03 | 1.11 |  | 1 | 0 | 2 | 1.17 | 1.15 |
| 15 | Angst vor einem Rückfall/einer Verschlechterung des Zustands | 1.92 | 1.26 |  | 2 | 1 | 3 | 0.32 | -1.05 |
| 16 | Einschränkungen aufgrund des Melanoms | 0.97 | 1.07 |  | 1 | 0 | 1 | 1.22 | 0.99 |
| 17 | Hoffnungsvoll in Bezug auf die Zukunft | 1.71 | 1.47 |  | 2 | 0 | 3 | 0.32 | -1.26 |
| 18 | Zufriedenheit mit den Informationen der Ärzte | 1.10 | 1.39 |  | 1 | 0 | 2 | 1.10 | -0.10 |
| 19 | Andere haben meine Krankheit nicht verstanden | 0.97 | 1.31 |  | 0 | 0 | 2 | 1.01 | -0.40 |
| 20 | Angst vor den Nachuntersuchungen | 1.71 | 1.35 |  | 2 | 1 | 3 | 0.25 | -1.17 |
| 21 | Sorgen aufgrund von Außenaktivitäten in der Sonne | 1.54 | 1.36 |  | 1 | 0 | 2 | 0.42 | -0.95 |
| 22 | Schuldgefühle, weil ich meinen Verpflichtungen nicht nachkommen kann | 0.71 | 1.16 |  | 0 | 0 | 1 | 1.55 | 1.23 |
| 23 | Unterstützung durch das Gesundheitssystem | 1.44 | 1.28 |  | 1 | 0.5 | 2 | 0.70 | -0.53 |
| 24 | Sorge um das Melanomrisiko von Verwandten | 1.61 | 1.30 |  | 1 | 1 | 3 | 0.33 | -1.10 |
| 25 | Unbehagen bei Intimität | 0.59 | 1.10 |  | 0 | 0 | 1 | 2.00 | 3.26 |
| 26 | Bedauern über frühere Sonnenexposition | 1.75 | 1.45 |  | 2 | 0 | 3 | 0.14 | -1.40 |
| 27 | Angst vor dem Tod | 1.66 | 1.38 |  | 1 | 1 | 3 | 0.48 | -1.07 |
| 28 | Sorge, dass die Bewältigung der täglichen Anforderungen nicht möglich ist | 1.08 | 1.25 |  | 1 | 0 | 2 | 1.04 | 0.04 |
| 29 | Störung durch Operationsnarbe | 0.80 | 1.23 |  | 0 | 0 | 1 | 1.44 | 0.85 |
| 30 | Verständnis der Behandlungsmöglichkeiten | 0.88 | 1.20 |  | 0 | 0 | 2 | 1.22 | 0.46 |
| 31 | Druck, die Haut zu überprüfen | 1.08 | 1.16 |  | 1 | 0 | 2 | 0.92 | 0.09 |
| 32 | Akzeptanz der Krankheit | 1.22 | 1.29 |  | 1 | 0 | 2 | 0.58 | -0.92 |
| 33 | Unterstützung durch Familie und Freunde | 0.97 | 1.39 |  | 0 | 0 | 2 | 1.18 | 0.00 |

Deskriptive Statistik der Items der 59 Teilnehmer, die vor der eigentlichen Rekrutierung in der Pilotstudie separat erhoben wurden (siehe auch ergänzende Abbildung S1)

Ergänzende Abbildung S1

Verteilung der EMQoLI-Items in den Pilotdaten.

Ergänzende Abbildung S2

Spearman-Korrelationen zwischen allen EMQoLI-Items im Pilotdatensatz (n = 59), die ein insgesamt positives Korrelationsmuster zeigen.

Ergänzende Abbildung S3

Spearman-Korrelationskoeffizienten zwischen allen in der Pilotstudie verwendeten Skalen (n = 59). Die Korrelationen zwischen der Summe aller EMQoLI-Items und den anderen Messinstrumenten entsprechen weitgehend den Zusammenhängen, die auch in der abschließenden Validierungsstudie (siehe Hauptmanuskript) beobachtet wurden: vergleichsweise geringe Zusammenhänge mit dem BIS, signifikant positive Korrelationen mit dem EORTC-Instrument sowie mit Angst und Depression, und eine negative Beziehung zur allgemeinen Selbstwirksamkeit.

|  |  |  |
| --- | --- | --- |

Essen Melanoma Quality of Life Inventory (EMQoLI)

Im Folgenden finden Sie eine Reihe von Aussagen zu verschiedenen Lebensbereichen, die durch Ihre Melanomerkrankung betroffen sein können. Bitte lesen Sie sich jede Aussage in Ruhe durch und beurteilen Sie, wie sehr (0 = gar nicht bis 4 = sehr stark) diese in den letzten 14 Tagen einschließlich heute auf Sie zutraf. Bitte bearbeiten Sie die Aussagen vollständig und lassen Sie keine aus.

|  | **Gar nicht** | **Ein wenig** | **Teil-weise** | **Etwas mehr** | **Sehr stark** |
| --- | --- | --- | --- | --- | --- |
| 1. Ich hatte Schmerzen im Bereich der Operationsnarbe des Melanoms und/oder der Metastasen. | ☐ 0 | ☐ 1 | ☐ 2 | ☐ 3 | ☐ 4 |
| 2. Ich hatte Schwellungen im Bereich der Operationsnarbe des Melanoms und/oder der Metastasen. | ☐ 0 | ☐ 1 | ☐ 2 | ☐ 3 | ☐ 4 |
| 3. Ich litt unter Müdigkeit. | ☐ 0 | ☐ 1 | ☐ 2 | ☐ 3 | ☐ 4 |
| 4. Ich litt unter Gelenkschmerzen. | ☐ 0 | ☐ 1 | ☐ 2 | ☐ 3 | ☐ 4 |
| 5. Ich litt unter Appetitlosigkeit. | ☐ 0 | ☐ 1 | ☐ 2 | ☐ 3 | ☐ 4 |
| 6. Ich litt unter Hautausschlag und/oder Juckreiz. | ☐ 0 | ☐ 1 | ☐ 2 | ☐ 3 | ☐ 4 |
| 7. Ich litt unter Übelkeit. | ☐ 0 | ☐ 1 | ☐ 2 | ☐ 3 | ☐ 4 |
| 8. Ich litt unter Erbrechen. | ☐ 0 | ☐ 1 | ☐ 2 | ☐ 3 | ☐ 4 |
| 9. Ich litt unter Durchfall. | ☐ 0 | ☐ 1 | ☐ 2 | ☐ 3 | ☐ 4 |
| 10. Ich litt unter Fieber. | ☐ 0 | ☐ 1 | ☐ 2 | ☐ 3 | ☐ 4 |
| 11. Ich litt unter weiteren Nebenwirkungen der Behandlung.   Weitere Nebenwirkungen: ____________________________________________  ____________________________________________ | ☐ 0 | ☐ 1 | ☐ 2 | ☐ 3 | ☐ 4 |
|  | **Gar nicht** | **Ein wenig** | **Teil-weise** | **Etwas mehr** | **Sehr stark** |
| 12. Ich fühlte mich durch die behandelnden Ärzte unterstützt. | ☐ 0 | ☐ 1 | ☐ 2 | ☐ 3 | ☐ 4 |
| 13. Ich hatte Angst vor einem Wiederauftreten der Erkrankung oder der Verschlechterung meines Zustandes. | ☐ 0 | ☐ 1 | ☐ 2 | ☐ 3 | ☐ 4 |
| 14. Ich habe hoffnungsvoll in die Zukunft geblickt. | ☐ 0 | ☐ 1 | ☐ 2 | ☐ 3 | ☐ 4 |
| 15. Ich war mit der Aufklärung durch die behandelnden Ärzte zufrieden. | ☐ 0 | ☐ 1 | ☐ 2 | ☐ 3 | ☐ 4 |
| 16. Es hat mich belastet, dass Andere (z.B. Arbeitskollegen, Freunde) die Schwere der Melanomerkrankung nicht erkennen. | ☐ 0 | ☐ 1 | ☐ 2 | ☐ 3 | ☐ 4 |
| 17. Ich hatte Angst vor dem Ergebnis der Folgeuntersuchungen. | ☐ 0 | ☐ 1 | ☐ 2 | ☐ 3 | ☐ 4 |
| 18. Ich hatte Bedenken, (Freizeit-)Aktivitäten in der Sonne nachzugehen (z.B. Spazierengehen, Sport, Urlaubsreisen). | ☐ 0 | ☐ 1 | ☐ 2 | ☐ 3 | ☐ 4 |
| 19. Ich fühlte mich vom Gesundheitssystem unterstützt (z.B. psychologische Unterstützung, Sozialdienst, Rehabilitationsangebot). | ☐ 0 | ☐ 1 | ☐ 2 | ☐ 3 | ☐ 4 |
| 20. Ich machte mir Sorgen um ein erhöhtes Melanomrisiko meiner Angehörigen. | ☐ 0 | ☐ 1 | ☐ 2 | ☐ 3 | ☐ 4 |
| 21. Ich hatte Angst, aufgrund meiner Erkrankung zu sterben. | ☐ 0 | ☐ 1 | ☐ 2 | ☐ 3 | ☐ 4 |
| 22. Die Narbe der Operation belastete mich. | ☐ 0 | ☐ 1 | ☐ 2 | ☐ 3 | ☐ 4 |
| 23. Ich hatte das Gefühl, die Behandlungsmöglichkeiten ausreichend gut verstanden zu haben. | ☐ 0 | ☐ 1 | ☐ 2 | ☐ 3 | ☐ 4 |
| 24. Ich empfand Druck, meine Haut ständig nach neuen Veränderungen (z.B. Rötungen, Leberflecke) untersuchen zu müssen. | ☐ 0 | ☐ 1 | ☐ 2 | ☐ 3 | ☐ 4 |
| 25. Ich fühlte mich durch mein privates Umfeld (z.B. Familie, Freunde) unterstützt. | ☐ 0 | ☐ 1 | ☐ 2 | ☐ 3 | ☐ 4 |

|  |  |  |
| --- | --- | --- |

Essen Melanoma Quality of Life Inventory (EMQoLI)

Für Behandler:innen – nicht für Patient:innen

Auswertung:

Höhere EMQoLI-Werte weisen auf eine stärkere Beeinträchtigung der gesundheitsbezogenen Lebensqualität hin.

Invertierte Fragebogen-Items: 12, 14, 15, 19, 23, 25

Skalen:

Symptome: Fragebogen-Items 3 – 11

Belastung: Fragebogen-Items 1, 2, 13, 16, 17, 18, 20, 21, 22, 24

Ressourcen: Fragebogen-Items 12, 14, 15, 19, 23, 25 (alle invertiert)
